# Lack of treatment options for endemic helminth infections in Chile affects patient care and public health

**DOI:** 10.1016/j.lana.2025.101131

**Published:** 2025-05-13

**Authors:** Thomas Weitzel, María Elvira Balcells, Claudia P. Cortes, Alberto Fica, Renzo Tassara, Jeannette Dabanch, Marisa Torres, Rafael Araos, Katia Abarca, Marcelo Wolff, Pablo Vial

**Affiliations:** a Programa Medicina del Viajero, Clínica Alemana, Facultad de Medicina Clínica Alemana, Universidad del Desarrollo, Santiago, Chile; b Instituto de Ciencias e Innovación en Medicina (ICIM), Facultad de Medicina Clínica Alemana, Universidad del Desarrollo, Santiago, Chile; c Institute of International Health, Charité Center for Global Health, Charité – Universitätsmedizin Berlin, Germany; d Departamento de Enfermedades Infecciosas del Adulto, Escuela de Medicina, Pontificia Universidad Católica de Chile, Santiago, Chile; e Departamento de Medicina Interna, Facultad de Medicina, Universidad de Chile, Santiago, Chile; f Fundación Arriarán, Santiago, Chile; g Servicio de Infectología, Hospital Base Valdivia, Valdivia, Chile; h Departamento de Pediatría y Cirugía Infantil, Facultad de Medicina, Universidad de Chile, Santiago, Chile; i Sección Infectología, Departamento de Medicina, Hospital Clínico de la Universidad de Chile, Santiago, Chile; j Escuela de Salud Pública, Pontificia Universidad Católica de Chile, Santiago, Chile; k Departamento de Enfermedades Infecciosas e Inmunología Pediátricas, Escuela de Medicina, Pontificia Universidad Católica de Chile, Santiago, Chile; l Chilean Academy of Medicine, Santiago, Chile

The untreatability of infectious diseases has a significant impact on patients’ physical and mental health but also causes stress and moral injury in frontline healthcare providers, as recently experienced during the COVID-19 pandemic.^1^^,^^2^

In case of neglected helminth infections, the situation is similar yet different. The armamentarium to treat nematodes, trematodes, and cestodes is well equipped and listed in the WHO List of Essential Medicines.^3^ Depending on the epidemiological situation, these drugs are used for mass treatment or individual therapy. The objectives are to prevent damage and long-term complications and to interrupt further spread of the parasite.

Chile is an emerging economy classified as a high-income country. The prevalence of soil-borne helminths (e.g. *Ascaris lumbricoides*) has decreased in recent decades in response to progress in hygiene and sanitation. However, other helminthic infections, including severe infections such as neurocysticercosis (NCC) and hydatid disease, remain endemic.^4^, ^5^, ^6^ Due to food habits, endemic fish-borne infections are emerging.^7^ Four different species of fish tapeworms are found in Chile, transmitted by the consumption of raw or marinated fish dishes, typically ceviche.^8^ Among these, *Dibothriocephalus latus* (formerly *Diphyllobothrium latum*) has the highest endemicity, infecting a significant proportion of food fish in the Lake District of southern Chile.^9^ The control of this invasive worm species is also urgently required under a One Health approach for its ecological and economic impact, threatening vulnerable native fish species.^9^

Praziquantel (PZQ) is widely used to treat and control schistosomiasis and to prevent NCC, and also remains the mainstay treatment for infections caused by other trematodes and adult cestode worms. PZQ is effective and cheap. In Chile, it is the only treatment option for the above-mentioned fish tapeworms, the beef tapeworm *Taenia saginata*, the pork tapeworm *T. solium*, and the dwarf tapeworm *Hymenolepis nana*; furthermore, PZQ is critical for combination therapy for NCC. Oral PZQ has been licensed in Chile by Merck since 1985, however, in 2019, the manufacturer decided to stop providing the relatively small Chilean market with PZQ. To compensate for this sudden deficit, the Chilean Ministry of Health (Department of Transmissible Diseases) purchased PZQ from Merck in Brazil and then received the drug as donations from the Pan American Health Organization (PAHO). PZQ was stocked in four public hospitals in the country and distributed free of charge through a ministerial order.^10^ Since the end of 2024, however, the PAHO donation has ended, and PZQ is no longer available in the country. Although the clinical course of intestinal tapeworm infections is mostly benign, the current situation is barely tolerable for patients, who suffer anxiety and distress from the blunt imagination of harbouring a gigantic worm in their body. Physicians, pharmacists, and other healthcare providers can only explain that treatment, consisting of a single dose of PZQ with a value of less than 5 USD, will be unavailable until further notice. The majority of the currently untreated cases are infected with fish tapeworms; however, there are also pork tapeworm carriers, who, by shedding infective eggs, pose an imminent risk to themselves and others of acquiring NCC, a devastating manifestation of this tapeworm's larval infection. Similar to past supply problems, the situation represents several concrete risks: physicians might resort to prescribing other ineffective anthelmintic drugs; patients may attempt self-treatment with veterinary PZQ formulations or seek to purchase PZQ abroad or through the internet. The latter leads to subsequent under-the-table distribution of leftover medication to other desperate patients, fueling self-medication attitudes and an incomplete treatment course that can contribute to parasitic resistance.

This letter aims to demonstrate a repetitive and multifactorial dilemma: 1. Pharmaceutical companies abandon low-profit products, regardless of the consequences for their clientele (patients, healthcare providers, public health); 2. Health authorities are legally restricted within a system of free markets, in which companies have little responsibility for the clinical consequences of their drug policies; 3. Media show limited or no interest in neglected parasitic diseases; and 4. Healthcare providers feel helpless and do not share their concerns about risks and damage caused by drug shortages. Unfortunately, the unavailability of certain pharmaceutical products in Chile also affects treatment options of various opportunistic infections affecting people living with HIV, and the management of non-endemic diseases, which are increasingly relevant for migrant populations and travelers, e.g. antimalarials and travel vaccines.

We feel that it is our responsibility to raise awareness of the current emergency, hoping that this might trigger joint efforts of manufacturers and national and international health authorities.

## Contributors

TW conceptualized the study, collected and curated data, and wrote the original draft. AF collected and curated data. RA and PV acquired funding. All authors analyzed and validated the data, and reviewed and edited the final draft.

## Declaration of interests

All authors declare no competing interests related to this work.
